# The Use of Patient-Generated Health Data From Consumer-Grade Mobile Devices in Clinical Workflows: Protocol for a Systematic Review

**DOI:** 10.2196/39389

**Published:** 2023-02-27

**Authors:** Sharon Guardado Medina, Minna Isomursu

**Affiliations:** 1 Empirical Software Engineering in Software, Systems and Services Faculty of Information Technology and Electrical Engineering University of Oulu Oulu Finland

**Keywords:** patient-generated health data, mobile health, mHealth, health care professional, mobile technology, health self-management, smartphone, mobile phone, condition, care, intervention, support, control, clinical, literature, data, devices, review, meta-analysis, PubMed, Scopus, integration, electronic, treatment

## Abstract

**Background:**

With the rapid advancement of mobile technology, the scope of mobile health (mHealth) has expanded to include consumer-grade devices such as smartphones and wearable sensors. These solutions have typically been used for fitness purposes; however, due to their ubiquitous capabilities for data collection, they have the potential to bridge information gaps and supplement data from clinical visits. Patient-generated health data (PGHD) can be derived from mHealth solutions and be used by health care professionals (HCPs) as complementary tools in the care process, yet their integration into clinical workflows presents a myriad of challenges. PGHD might be a new and unfamiliar source of information for most HCPs, and the majority of mHealth solutions have not been designed to be used by HCPs as active reviewers. As mHealth solutions become more available and attractive to patients, HCPs may see an increase in the influx of data and related inquiries from their patients. This mismatch in expectations can result in disruptions to clinical workflows and negatively impact patient-clinician relationships. For PGHD to be integrated into clinical workflows, its use should be proven beneficial for both patients and HCPs. However, so far, only limited research has been done on the concrete experiences of HCPs as active reviewers of PGHD from consumer-grade mobile devices.

**Objective:**

We aimed to systematically guide the review of existing literature to identify what types of PGHD from consumer-grade mobile devices are currently being used by HCPs as complementary tools in the care process.

**Methods:**

The PRISMA-P (Preferred Reporting Items for Systematic Review and Meta-Analysis Protocols) 2015 was followed for the design of the search, selection, and data synthesis processes. Electronic searches will be done on PubMed, ACM Digital Library, IEEE Xplore, and Scopus.

**Results:**

Preliminary searches have been conducted, and previous related systematic and scoping reviews have been found and evaluated. The review is expected to be completed in February 2023.

**Conclusions:**

This protocol will guide the review of existing literature on the use of PGHD produced by consumer-grade mobile devices. Although there have been previous reviews related to this topic, our proposed approach seeks to understand the specific opinions and experiences of different types of HCPs who are already using PGHD in their clinical practice and the motives for deeming these data useful and worth reviewing. Depending on the studies that will be included, there may be an opportunity to provide a wider understanding of what types of HCPs trust PGHD, despite the possible challenges that its use might convey, potentially contributing with the knowledge to support the design strategies of mHealth tools that could be integrated into clinical workflows.

**International Registered Report Identifier (IRRID):**

PRR1-10.2196/39389

## Introduction

### Background

Mobile health (mHealth) refers to the use of mobile technology to obtain data pertinent to wellness and disease diagnosis, prevention, and management [1]. With the rapid advancement of mobile technology in the last decade, the scope of mHealth has expanded to include consumer-grade devices, such as smartphones and wearable sensors, which allow for convenient access to health apps [2], motion sensing, as well as physiological and biochemical tracking. The certification of medical-grade mobile devices is based on clinical evidence, often needing years to bring a device to the market [3]; however, consumer-grade devices are evolving at a more rapid pace, and although they are typically used for fitness purposes, their capabilities for data collection are opening possibilities for their use in the medical sphere as well [4]. Ubiquitous health data collection can theoretically make it possible to monitor and intervene whenever and wherever acute and chronic medical conditions occur [1].

The data created, recorded, and gathered by and from patients, using mHealth tools such as smartphones and wearable devices, are known as patient-generated health data (PGHD) [5]. The PGHD derived from mHealth solutions has the potential to bridge information gaps and supplement data from clinical visits by providing a rich picture of a patient’s daily behaviors, environment, lifestyle, and biometric data outside of the clinic.

In the past, mHealth solutions have been mostly designed for general wellness purposes; in recent years, mHealth interventions targeting specific health conditions have been increasing [6]. Likewise, the body of evidence that proves the potential value of mHealth is growing [7]; however, there seems to be only limited evidence of its efficacy [8]. Furthermore, the strength and quality of clinical evidence have been identified as factors that impact the adoption of mHealth tools by health care professionals (HCPs). Although the adoption of HCPs seems to be one of the most influential factors regarding the success of mHealth tools [7], there has not been much research on the role HCPs are expected to play in the implementation process of mHealth tools [3,9].

The use of mHealth tools by patients enables access to electronic PGHD by HCPs; however, access does not equate to active use. Furthermore, the integration of PGHD into clinical workflows presents a myriad of challenges, including data security and privacy, data standardization, difficult and time-consuming interpretation, and interoperability, among others [5,10].

In regular clinical workflows, patients often interact with HCPs with different medical specialities, who might have different approaches and goals when collecting data from their patients, depending on their role in the care process. A scoping review by Nittas et al [9] identified that there are 2 roles that HCPs can take when integrating PGHD into the care process: the supporter and the reviewer. In the supporter role, the HCP mostly motivates patients and oversees the use of PGHD. In the reviewer role, the HCP analyzes PGHD to personalize advice, conduct remote monitoring, and complement medical data [9]. PGHD can encompass diverse data sets that come from both passive sensing (through mobile and wearable devices) and active sensing (through mHealth apps) [1]; therefore, PGHD might be a new and unfamiliar source of information for most HCPs [11]. Considering that the reviewing tasks imply a more active stance from HCPs, it is reasonable to assume that not all of them might be interested or confident in becoming PGHD reviewers.

mHealth solutions are becoming increasingly available and attractive to patients, with many integrating mHealth apps and tracking devices into their everyday life as ways to improve their health and well-being [12]. A recent scoping review identified 31 studies that evaluated mHealth interventions for self-management of chronic conditions [3], of which over a third required or encouraged patients to share data with their health care providers. However, only 2 of those studies described mHealth solutions that were meant to be used by HCPs as active reviewers. This sort of mismatch in expectations from patients and HCPs can result in disruptions to existing clinical workflows and negatively impact patient-clinician relationships [13].

For PGHD to be effectively integrated into clinical workflows as a complementary tool to bridge information gaps and supplement data from clinical visits, their use should prove beneficial for both patients and HCPs. However, so far, little research has been done on the concrete experience of HCPs as active reviewers of PGHD from consumer-grade mHealth solutions.

### Objectives

The main objective of our review will be to systematically analyze existing literature to try to identify the profile of HCPs who are using PGHD from consumer-grade mobile devices as complementary tools for their clinical practices.

The secondary objective will include the identification of the types of PGHD, produced by consumer-grade mobile devices and mHealth apps, that HCPs might deem useful and worth reviewing.

To attain these objectives the research questions to be addressed by the review are as follows:

- How are the PGHD from consumer-grade mobile devices used by HCPs in the clinical workflow?
- Which types of HCPs are actively reviewing PGHD in their clinical practice?
- What kind of PGHD from consumer-grade devices do HCPs find useful?

## Methods

### Design

For the design of this protocol, the PRISMA-P (Preferred Reporting Items for Systematic Review and Meta-Analysis Protocols) 2015 [14] was followed. For the design of the search, selection, and synthesis processes, the guidelines for performing systematic literature reviews in software engineering [15] were also consulted.

For the review design, we will use a convergent design for systematic mixed studies reviews [16]. The systematic review will adhere to the PRISMA (Preferred Reporting Items for Systematic Reviews and Meta-Analyses) statement [17].

### Search Strategy

Eligible studies will be identified through comprehensive literature searches conducted in the bibliographical databases that focus on health and biomedicine, information technology domains, or encompass journal articles and conference proceedings from multiple disciplines. The selected sources are PubMed, ACM Digital Library (including the ACM Guide to Computing Literature), IEEE Xplore, and Scopus. The advanced search methodologies and limitations of each source were revised in advance to standardize the final search terms and resulting search string, which will be used in all sources without alterations to maintain consistency.

The search strategy was constructed iteratively to ensure that relevant articles were more likely to be identified. After a pilot search in PubMed, it was deemed that the searches should be delimited by 3 key topics: “patient-generated health data,” “health personnel,” and “mobile technologies.” Considering that the key aspects of this review will be more relevant to the medical context than to the technological context, the Medical Subject Headings will be used to search for literature.

Terms comparable to “patient-generated health data,” “health personnel,” and “mobile technologies” were added to the search string using AND operators. To avoid overlooking relevant articles by excluding terms and to prevent retrieving an excessive number of irrelevant results, exact phrases were used with some of the terms. To identify the most appropriate phrases, trial searches were performed in Google Scholar, using possible variations identified in the literature. The final search string was validated by a research librarian from the University of Oulu (Table 1).

Additional filters will be used in all sources to limit the results to articles published in scientific peer-reviewed journals or conferences, written in English, with a publication date between January 2013 and May 2022, and with topics related to medical disciplines or the field of information technologies.

Once the electronic searches have been completed, a supplementary hand search of studies will be performed within previous systematic literature reviews and scoping reviews related to the topic PGHD that were identified in the pilot searches.

**Table 1:** Search string to be used in the electronic searches.

Concept	Search string	Definition
“Patient-generated health data”	“Patient-generated health data” OR “patient health data” OR “patients health data” OR “patient-generated data” OR “person-generated data” OR “person-generated health data” OR “patient-reported data” OR “patient self-reported data” OR “patients self-reported data” OR “patient-generated clinical data” OR “patient-generated medical data” OR “self-reported patient data” OR “self-generated patient data” OR “personal health data” OR “mHealth patient data” OR “patient collected data” OR “patient-collected health data” OR “self-collected health data” AND	MeSH^a^ terms introduced in 2018 to refer to health-related data created, recorded, or gathered (outside of clinical settings) by patients, family members, or caregivers to help address a health concern [18].
“Health personnel”	“Health personnel” OR “care professionals” OR “medical professionals” OR “health care professionals” OR “health professionals” OR ”health care providers” OR “health providers” OR “medical providers” OR “health care worker” OR “health care workers” OR “health care worker” OR “health care workers” OR practitioners OR specialists OR clinicians OR doctors OR nurses OR physicians OR physiotherapists OR “clinical team” OR “clinical staff“ OR “medical staff” OR “medical team” AND	MeSH terms in use since 1992 to refer to people working in the provision of health services, whether as individual practitioners or employees of health institutions and programs [19].
“Mobile technologies”	“Mobile technolog*” OR “mobile phone” OR “mobile device” OR “mobile phones” OR “mobile devices” OR “smart device” OR smartphone OR smartwatch OR “smart ring” OR “smart device” OR “smart devices” OR wearable* OR “health tracker” OR “activity tracker” OR “fitness tracker” OR “mobile app*” OR “health app*” OR “mobile health intervention” OR “mHealth intervention” OR “mobile health solution” OR “mHealth solution” OR “mHealth solutions” OR “mobile health tool” OR “mHealth technology”	These terms commonly refer to internet-enabled devices like smartphones, tablets, and smartwatches. These are the latest in a progression that includes two-way pagers, notebook computers, mobile telephones, GPS navigation devices, and more [20].

^a^MeSH: Medical Subject Heading.

### Inclusion Criteria

#### Types of Studies

Primary studies will be included irrespective of their design. Both qualitative and quantitative research findings from the selected studies will be integrated, with the use of qualitative data transformation technique methods [21]. Eligible studies will include clinical trials, nonrandomized controlled trials, cross-sectional studies, longitudinal studies, observational studies, case studies, and other types of qualitative studies. The studies must have been published in peer-reviewed scientific journals and conferences.

#### Types of Participants

The review will focus on the experiences of HCPs who have already used PGHD in their clinical practice, as part of standalone mHealth interventions, by personal initiative, or for any other reasons. The type of HCP is not being limited to specific medical specialities as long as the participants can be recognized as health care workers who are interacting directly with patients.

#### Types of Technology

The review will focus on mHealth solutions that allow for patient data to be collected outside of clinical settings by patients, using mobile apps or commercial mobile devices such as smartphones or tablets and wearable devices like smartwatches, smart rings, fitness bands, chest straps, and other wearable health trackers.

The current mobile and wearable technologies started to become more available to consumers at the beginning of the last decade. As of 2013, only a few mHealth interventions that aimed at assessing the impact of native apps for smartphones had been reported in the literature, all of which had been created only for research purposes and were not yet available in public app stores [2]; therefore, our search will be limited to articles published since 2013.

### Exclusion Criteria

- The articles that explicitly indicate they are study protocols, scoping reviews, and systematic literature reviews will automatically be excluded.
- Studies on interventions based on SMS, chats, social media interactions, phone calls, or another type of two-way communications as the main method for data collection will not be included; we did not consider them to be mHealth solutions, as they are targeted for communication in general and require additional time and effort from HCPs not only to review data but also to maintain the communication between appointments.
- Studies based on interventions that required the use of specialized medical-grade devices that are not designed to be worn or meant to be used as mobile trackers will not be included.
- Studies related to nonelectronic PGHD.

### Data Collection and Analysis

#### Selection of Studies

After the electronic search in all sources and the supplementary hand search of systematic reviews are finalized, the resulting articles will be imported into Covidence (Veritas Health Innovation) to conduct the screening process. Covidence will automatically identify duplicates and allows for multiple reviewers to perform the screening and selection process in a consensual manner.

The initial screening will be limited to the titles and abstracts. This part of the process will be done independently by 2 reviewers to reduce bias. Both reviewers in charge of this stage have a background in computer science and previous research experience with mHealth and PGHD. Before starting the screening process, the reviewers will complete a joint exercise to validate the reviewing methodology and ensure that both reviewers understand the inclusion and exclusion criteria correctly.

Once the reviewers have a preliminary list of candidate studies to be included, they will proceed with the full-text screening of those articles.

During the full-text screening, reviewers will also try to identify if multiple papers could be based on the same study. In this case, both reviewers will revise the possibly duplicated articles and decide, based on author names, study descriptions, and sample characteristics. In the case of duplications, the most recent article would be preferred; however, the final decision should be agreed upon by both reviewers, considering which papers better fits the inclusion criteria.

In case of disagreement in either the initial screening or during the full-text screening, both reviewers will discuss the reasons for disagreement. In case the disagreement persists, a third reviewer will act as an arbitrator and will review the article to decide whether or not the article should be included. The disagreements will be documented, and the reason for the final inclusion or exclusion will be recorded.

The search and screening results will be reported using a PRISMA flow diagram.

#### Data Extraction

The key information of the included articles will be extracted using the Data Extraction Form function offered in Covidence. The extraction task will be completed by the 2 initial reviewers and 2 additional reviewers, all of whom have previous research experience with the topics related to this review.

Each article will be assigned randomly to 2 reviewers. Each reviewer will extract the data independently and the data extracted by both reviewers will be compared using Covidence. In the case of differences in the extracted data, a consensus will be reached between the 2 primary reviewers. In the case of differences that could not be agreed upon by the 2 primary reviewers, the conflicting data will be discussed by the 4 reviewers to reach a consensus.

The data extraction form will assist in the extraction of relevant information from the selected studies, following the template in Table 2. The main author will pilot the extraction of 5 articles, using the proposed extraction form, to identify possible adjustments that could be required before the data extraction process starts with the reviewing team.

In the case of papers that do not report complete descriptions of the intervention, outcomes, or other information relevant to this research, the article author will be contacted via email to ask for complementary information.

**Table 2:** Basic template of the data extraction form.

Item	Characteristics
Article general information	Year of publication Author(s)’ name(s) Country of origin
Characteristics of the study	Objectives Main findings
Research methods	Sampling method Sample size Data collection method
Information about participants	Profession or medical specialty Main motivation of HCPs^a^ to use PGHD^b^
Context of PGHD use	Conditions being treated Mobile technology used Type of PGHD collected The mechanism for PGHD visualization

^a^HCP: health care professional.

^b^PGHD: patient-generated health data.

#### Studies’ Quality Assessment

The quality of included studies will be assessed in parallel to the data extraction process, using a quality assessment form that will be included in Covidence, following the checklist to assess the quality of studies proposed by Kitchenham [15].

#### Data Synthesis

The included studies might comprise qualitative and quantitative data that will need to be transformed into a qualitative format and be synthesized through narrative synthesis. For this, we will be using a descriptive narrative form with an appropriate table format. The extracted data will be organized in the table by type of technology, type of condition, type of participants, and type of data being collected or used to identify any interesting findings, trends, relationships, and limitations that can assist in addressing the proposed review questions.

A data coding strategy is suggested for the analysis. NVivo (QSR International) will be used to assist in the data coding strategy. The codes will be defined in accordance with the research objectives of the review. The strategy will be piloted with 5 studies, and any necessary changes to the strategy will be made and later reported in the full review.

## Results

As of May 2022, preliminary searches have been done to refine the search strategy and assess the availability of studies on our research topic. Previous related systematic and scoping reviews have been found and evaluated. Systematic searches, data extraction, data synthesis, analysis, and the writing of the systematic review document are expected to be completed by February 2023.

## Discussion

### Expected Outcomes

The proposed systematic review will report on the use of PGHD produced by consumer-grade mobile technology, as complementary tools for HCPs in the clinical workflow. Considering that most research related to this topic has been done under controlled conditions, our interest lies in identifying the characteristics of PGHD that foster its use by HCPs. We assume these characteristics might comprise aspects such as the type of clinical activities where PGHD from consumer-grade devices could be useful and the particular characteristics of HCPs who are willingly using PGHD due to the realization of PGHD benefits to complement their current practices.

Previous research on PGHD has explored its effect on patient-clinician relationships [13], its use in clinical practices [11], and its use to facilitate prevention and health promotion [9]. Nevertheless, after examining these reviews, we have identified 3 determinant aspects for our review that will report on research gaps that previous reviews have not addressed in detail. The first aspect is that previous reviews did not limit their research to PGHD collected outside of the clinic but also included other types of PGHD as patient-reported outcomes collected during appointments with HCPs [13]. Second, the types of mobile technologies from which PGHD has been produced have not been limited to consumer-grade mobile or wearable devices but have also included medical-grade devices [9,11,13], which tend to be more accurate and more widely accepted in the medical field. The last differentiating aspect is that previous research has not focused on understanding the specific experiences of HCPs who are already performing the role of PGHD reviewers to support their patients’ care but mostly on their expectations as potential users or in a predominantly supportive role.

The use of PGHD is a relatively new trend, and its relevance might be different across countries, medical specialities, or clinical settings. Depending on the studies that will be included in this review, there may be an opportunity to provide a wider understanding of what types of HCPs trust PGHD from consumer-grade mobile solutions despite the potential challenges that its use might convey. We are interested in understanding why and how they have decided to use this type of data, assuming this knowledge could contribute to the design strategies of mHealth tools that could be integrated into clinical workflows.

### Limitations

Some limitations to the design of this review include the exclusion of papers not written in English and considering only papers that explicitly mention PGHD (or its possible variations). PGHD is a relatively new definition, and it could be possible that some relevant papers on mHealth interventions might not explicitly address PGHD using that specific term but instead could have used other terms not included in our search string.

## Abbreviations

HCP: health care professional
mHealth: mobile health
PGHD: patient-generated health data
PRISMA-P: Preferred Reporting Items for Systematic Review and Meta-Analysis Protocols
PRISMA: Preferred Reporting Items for Systematic Review and Meta-Analyses
